# CRISPR-Cas9 mediated gene deletions in lager yeast *Saccharomyces pastorianus*

**DOI:** 10.1186/s12934-017-0835-1

**Published:** 2017-12-05

**Authors:** Arthur R. Gorter de Vries, Philip A. de Groot, Marcel van den Broek, Jean-Marc G. Daran

**Affiliations:** Department of Biotechnology, Delft University of Technology, Van der Maasweg 9, 2629 HZ Delft, The Netherlands

**Keywords:** *Saccharomyces pastorianus*, Brewing, CRISPR-Cas9, Genome editing, Ribozymes

## Abstract

**Background:**

The ease of use of CRISPR-Cas9 reprogramming, its high efficacy, and its multiplexing capabilities have brought this technology at the forefront of genome editing techniques. *Saccharomyces pastorianus* is an aneuploid interspecific hybrid of *Saccharomyces cerevisiae* and *Saccharomyces eubayanus* that has been domesticated for centuries and is used for the industrial fermentation of lager beer. For yet uncharacterised reasons, this hybrid yeast is far more resilient to genetic alteration than its ancestor *S. cerevisiae*.

**Results:**

This study reports a new CRISPR-Cas9 method for accurate gene deletion in *S. pastorianus*. This method combined the *Streptococcus pyogenes cas9* gene expressed from either a chromosomal locus or from a mobile genetic element in combination with a plasmid-borne gRNA expression cassette. While the well-established gRNA expression system using the RNA polymerase III dependent *SNR52* promoter failed, expression of a gRNA flanked with Hammerhead and Hepatitis Delta Virus ribozymes using the RNA polymerase II dependent *TDH3* promoter successfully led to accurate deletion of all four alleles of the *SeILV6* gene in strain CBS1483. Furthermore the expression of two ribozyme-flanked gRNAs separated by a 10-bp linker in a polycistronic array successfully led to the simultaneous deletion of *SeATF1* and *SeATF2*, genes located on two separate chromosomes. The expression of this array resulted in the precise deletion of all five and four alleles mediated by homologous recombination in the strains CBS1483 and Weihenstephan 34/70 respectively, demonstrating the multiplexing abilities of this gRNA expression design.

**Conclusions:**

These results firmly established that CRISPR-Cas9 significantly facilitates and accelerates genome editing in *S. pastorianus*.

## Background

Lager beer is the most produced fermented beverage: in 2015 the worldwide production reached a global volume of 170 × 10^+9^ L. The fermentation workhorse of lager brewing is *Saccharomyces pastorianus*, a natural interspecific hybrid of *Saccharomyces cerevisiae* and *Saccharomyces eubayanus* [1, 2] whose domestication is thought to have occurred in central Europe (Bohemia, nowadays Czech republic) in the late Middle Ages. Its ability to ferment at low temperature, to flocculate and to produce a vast range of flavour compounds make *S. pastorianus* well suited for the brewing process. In addition to their hybrid nature, *S. pastorianus* strains share a high degree of aneuploidy. While the first strain of *S. pastorianus* Weihenstephan 34/70 was sequenced in 2009 [2], the exact chromosome complement of lager yeast was revealed later with the introduction of next generation sequencing [3–6]. Within *S. pastorianus* genomes, chromosomes may be completely absent or present in up to five copies and chromosome copy numbers vary widely across different strains [4]. This intricate genome organisation significantly complicates functional gene analysis. Indeed, a simple gene deletion based on double crossover mediated by homologous recombination requires successive removal of all copies of the gene in both sub-genomes by several rounds of transformation. In association with a low propensity to perform homologous recombination, the difficulty to delete high copy number genes may explain the quasi-absence of examples of functional characterisation of *S. pastorianus* genes in the scientific literature based on impact of gene deletion [7–9]. Instead a *S. pastorianus* gene or allele is usually cloned in *S. cerevisiae* and characterised based on the impact of the overexpression. However, such approaches do not take into account the role of the orthologous gene harboured by the other sub-genome, the possible occurrence of paralogs, and the gene expression regulation of the gene in its allo-aneuploid genetic background. Therefore, tools are needed to achieve efficient genome editing in allo-aneuploid *S. pastorianus* not only to enable targeted genetic modification, but also to enable functional gene analysis.

The exposed DNA strand ends resulting from a DNA double strand break (DSB) are extremely recombinogenic [10, 11]. Even in *Saccharomyces cerevisiae* that exhibits a natural inclination to perform homologous recombination, introduction of a programmed DSB by combining the insertion an I-*Sce*I restriction site in a chromosomal locus and expression of the endonuclease encoding gene *SCEI* showed substantial stimulation of homologous recombination at the cut site enabling the correct assembly of multiple DNA fragments [12]. Although efficient, the use of *Sce*I induced DSB is limited since it requires the insertion of the recognition site prior its utilisation. In the past 5 years, the advent of the CRISPR (clustered regularly interspaced short palindromic repeat)—Cas9 (CRISPR-associated protein 9) system derived from *Streptococcus pyogenes* has considerably transformed genome engineering approaches [13, 14]. The system comprises two elements: a short chimeric RNA that derives from the fusion of the tracr and crRNA called guide RNA (gRNA), and the endonuclease Cas9 [13, 14]. By forming a complex with Cas9, the gRNA provides sequence specificity to the system. The hetero-duplex formed by the gRNA and the genomic target places the endonuclease which generates a blunt ended DSB. The systems has been successfully implemented in *S. cerevisiae* [15–19], which broadened genome editing possibilities by allowing multiplexing [15, 16, 18] and high precision in vivo site-directed mutagenesis [15]. The expression of the gRNA has been a point of attention since the gRNA secondary structures are crucial for the formation of the complex with Cas9. Therefore the 5′ capping and 3′ polyadenylation present in RNA polymerase II transcripts have to be avoided. By analogy with the expression of gRNA in human cell lines [14], placing the gRNA behind the control of a RNA polymerase III dependent promoter (e.g. *SNR52*p) resulted in expression of an active gRNA lacking these modifications [16]. In addition, due to the lack of polyadenylation-mediated export to the cytosol, RNA polymerase III transcribed gRNAs reside in the nucleus longer where they can form a complex with Cas9.

However gRNA expression from a RNA polymerase III was shown to result in low and unstable transcript levels [20]. To overcome this issue while avoiding inactivation of the gRNA by 5′ capping and 3′ polyadenylation, the gRNA can be flanked by two ribozymes molecules and expressed by RNA polymerase II. Upon transcription the ribozymes self-cleave, resulting in removal of 5′ and 3- ends and release of a mature gRNA [19, 21]. Such CRISPR-Cas9 systems have been confirmed to mediate efficient genome editing in multiple cell types already, such as human cell lines [13, 14, 22], mice [23], zebrafish [24], *Caenorhabditis elegans* [25, 26], *Drosophila* [27], yeasts [15, 16, 28, 29], and plants [30–32].

The goal of the present study was to explore the use of CRISPR-Cas9 in *S. pastorianus,* a yeast with low genetic accessibility that is characterised by a unique allo-aneuploid genome. To this end, we present the construction of molecular tools to achieve efficient single and double simultaneous gene deletions. The successful application of this methodology offers an opportunity to get a deeper understanding of hybrid yeast biology.

## Methods

### Strains and growth conditions

**Fig. 1:**
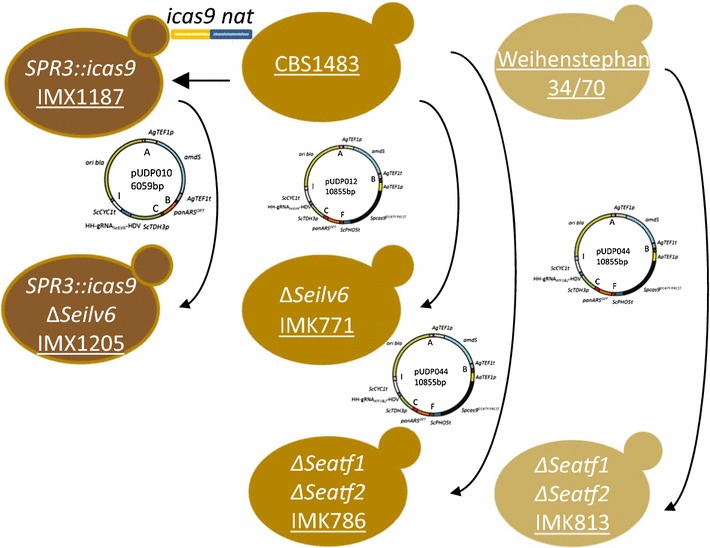
Strains construction flow-chart. Schematic representation of the different strain lineages constructed in this study. The strain name is underlined and each arrow indicates a transformation step

The *S. pastorianus* and *cerevisiae* strains used in this study are listed in Table 1 and a construction flow-chart is provided in Fig. 1.

**Table 1:** Strains used throughout this study

Name	Species	Genotype	Source
CBS1483	*S. pastorianus*	Wildtype	[4]
IMX1187	*S. pastorianus*	*SPR3::AaTEF1p*-*Spcas9* ^*D147Y P411T*^-*ScPHO5t*	This study
IMX1205	*S. pastorianus*	*SPR3::AaTEF1p*-*Spcas9* ^*D147Y P411T*^-*ScPHO5t* Δ*Seilv6*	This study
IMK771	*S. pastorianus*	Δ*Seilv6*	This study
IMK786	*S. pastorianus*	Δ*Seatf1* Δ*Seatf2*	This study
Weihenstephan 34/70	*S. pastorianus*	Wildtype	[2, 66]
IMK813	*S. pastorianus*	Δ*Seatf1* Δ*Seatf2*	This study
CEN.PK113-7D	*S. cerevisiae*	*MAT*a *MAL2*-*8c*	[67]
IMX585	*S. cerevisiae*	*MAT*a *can1Δ::AaTEF1p*-*Spcas9* ^*D147Y P411T*^-*ScPHO5t* natNT2	[15]

Under nonselective conditions, *Saccharomyces pastorianus* and *cerevisiae* strains were grown in complex medium (YPD) containing 10 g L^−1^ yeast extract, 20 g L^−1^ peptone, and 20 g L^−1^ glucose. For nourseothricin selection, YPD medium was supplemented with 100 μg L^−1^ of the antibiotic. Synthetic media (SM) containing 20 g L^−1^ glucose, 3 g L^−1^ KH_2_PO_4_, 0.5 g L^−1^ MgSO_4_7H_2_O, 5 g L^−1^ (NH_4_)_2_SO_4_, 1 mL L^−1^ of a trace element solution and of a vitamin solution was prepared as previously described [33]. For selection of yeast strains harboring an acetamidase marker [34] (NH_4_)_2_SO_4_ was replaced by 0.6 g L^−1^ acetamide as nitrogen source and 6.6 g L^−1^ K_2_SO_4_ to compensate for sulfate (SM-Ac). Loss of the acetamide marker was selected for on SM containing 2.3 g L^−1^ fluoroacetamide (SM-Fac) [34]. The pH in all media was adjusted to 6.0 with KOH. Solid media were prepared by adding 2% agar to the various media. The strains of *S. pastorianus* and *cerevisiae* were incubated at 20 and 30 °C respectively.

Shake flask cultures of *S. pastorianus* were grown at 20 °C in 500 mL flasks containing 100 mL complete medium (YPD) with 20 g L^−1^ glucose in an Innova 43/43R shaker (Eppendorf, Hauppauge, NY) set at 200 rpm. Frozen stocks were prepared by addition of glycerol (30% v/v) to exponentially growing shake-flask cultures of *S. cerevisiae*, *S. pastorianus* and overnight cultures of *Escherichia coli* and stored aseptically in 1 mL aliquots at − 80 °C.

For growth studies in shake flasks, *S. pastorianus* strains were grown in shake flasks with complete medium YPD. Growth rates were based on optical density at 660 nm (OD_660_) measurements using a Libra S11 spectrophotometer (Biochrom, Cambridge, United Kingdom). Specific growth rates were calculated from exponential fits of the OD_660_ against time.

### Plasmid construction

All plasmids and primers used during this study are shown in Tables 2 and 3, respectively. The DNA parts harboured by the plasmids pUD527, pUD528, pUD530, pUD531, pUD532 pUD536 and pUD573 were de novo synthesised at GeneArt (Thermo Fisher Scientific, Waltham, MA). Unless specified, plasmids were propagated and stored in *E. coli* strain XL1-blue. Yeast transformation was done by electroporation using 50 µL of competent cells and up to 5 µL DNA as previously described [35] and transformed cells were incubated in 0.5 mL YPD during 1 h, after which they were re-suspended in 100 µL of sterile demi-water and plated on selective medium. High fidelity PCR amplification was performed using Phusion polymerase (Thermo Fisher Scientific) according to supplier’s instructions.

**Table 2:** Plasmids used throughout the study

Name	Relevant genotype	Source	Addgene ID#^a^
pCT	*ori amp* ^*r*^ *ARS4 CEN6 LEU2 AaTEF1p*-*Spcas9* ^*D147Y P411T*^-*ScPHO5*	[36]	
pMEL15	*ori amp* ^*r*^ 2 μm *natNT2 SNR52*p-gRNA_*CAN1*_-*SUP4t*	[15]	
pROS12	*ori amp* ^*r*^ 2 μm *hphNT1* gRNA _*CAN1*_ gRNA _*ADE2*_	[15]	
pUC19	*ori amp* ^*r*^ LacZ multiple cloning site	[68]	
pUD423	*ori amp* ^*r*^ *AaTEF1p*-*Spcas9* ^*D147Y P411T*^-*ScPHO5t*	GeneArt™	
pUD526	*ori amp* ^*r* NotI^ HRL *AaTEF1p*-*Spcas9* ^*D147Y P411T*^-*ScPHO5t natNT2* HRM^NotI^	GeneArt™	
pUD527	*ori kan* ^*r*^ SHRA *AgTEF1p*-*amdS*-*AgTEF1t* SHRB	GeneArt™	
pUD528	*ori kan* ^*r*^ SHRB *AaTEF1p*-*Spcas9* ^*D147Y P411T*^-*PHO5t*	GeneArt™	
pUD530	*ori kan* ^*r*^ SHRB panARSopt SHRC	GeneArt™	
pUD531	*ori kan* ^*r*^ SHRC *TDH3p* ^BsaI BsaI^ *CYC1t* SHRI	GeneArt™	
pUD532	*ori kan* ^*r*^ SHRI *bla ori* SHRA	GeneArt™	
pUD536	*ori amp* ^*r* BsaI^ gRNA_SeILV6_^BsaI^	GeneArt™	
pUD573	*ori amp* ^*r* BsaI^ gRNA_SeATF1_ gRNA_SeATF2_^BsaI^	GeneArt™	
pUDP003	*ori amp* ^*r*^ panARSopt *AgTEF1p*-*amdS*-*AgTEF1t TDH3p* ^BsaI BsaI^ *CYC1t*	This study	101164
pUDP004	*ori amp* ^*r*^ panARSopt *AgTEF1p*-*amdS*-*AgTEF1t TDH3p* ^BsaI BsaI^ *CYC1t AaTEF1p*-*Spcas9* ^*D147Y P411T*^-*ScPHO5t*	This study	101165
pUDP010	*ori amp* ^*r*^ panARSopt *AgTEF1p*-*amdS*-*AgTEF1t TDH3p*-HH-gRNA_*SeILV6*_-HDV-*CYC1t*	This study	101166
pUDP012	*ori amp* ^*r*^ panARSopt *AgTEF1p*-*amdS*-*AgTEF1t TDH3p*-HH-gRNA_*SeILV6*_-HDV-*CYC1t AaTEF1p*-*Spcas9* ^*D147Y P411T*^-*ScPHO5t*	This study	101167
pUDP044	*ori amp* ^*r*^ panARSopt *AgTEF1p*-*amdS*-*AgTEF1t TDH3p*-HH-gRNA_*SeATF1*_ -*HDV*-*HH*-*gRNA*_SeATF2_-HDV-*CYC1t* *AaTEF1p*-*Spcas9* ^*D147Y P411T*^-*ScPHO5t*	This study	101168
pUDR107	*ori amp* ^*r*^ 2 *μ*m *hphNT1* gRNA_*URA3*_	This study	

HRL and HRM indicate the left and right homology arms for integration on the *SPR3* locus, SHR stands for synthetic homologous recombination sequence and enzyme digestion sites are indicated in superscript

^a^ https://www.addgene.org/

**Table 3:** Primers used in this study

Name	Sequence	Purpose
3274	TATTCACGTAGACGGATAGGTATAGC	Amplification SHR I
3275	GTGCCTATTGATGATCTGGCGGAATG	Amplification SHR A
3276	GTTGAACATTCTTAGGCTGGTCGAATC	Amplification SHR B
3283	ACGTCTCACGGATCGTATATGC	Amplification SHR C
3597	ATTAAGGGTTCTCGAGAGC	Amplification *natNT2*
3750	GAGGCGTTAGTTTGGCTAATGAG	Diagnostic primer
3841	CACCTTTCGAGAGGACGATG	Amplification SHR B
3847	ACTATATGTGAAGGCATGGCTATGG	Amplification SHR A
3856	CTAGCGTGTCCTCGCATAGTTC	Amplification SHR C
4068	GCCTACGGTTCCCGAAGTATGC	Amplification SHR I
6005	GATCATTTATCTTTCACTGCGGAGAAG	pROS12 backbone
7389	GGTTTCTTAGACGTCAGGTGGC	pUC19 backbone
8076	GTTTAGCTCTATGGTGCAAAATTCTCCAGAAAAAAGGGATCCATAGAAAAGAATATGTCTAATTGAAAAATAGATATGTACCATAAGTAAAGTGCATGCGTGTATACCGAAACCAAGACA	Repair DNA SeURA3
8077	TGTCTTGGTTTCGGTATACACGCATGCACTTTACTTATGGTACATATCTATTTTTCAATTAGACATATTCTTTTCTATGGATCCCTTTTTTCTGGAGAATTTTGCACCATAGAGCTAAAC	Repair DNA SeURA3
8314	TGCGCATGTTTCGGCGTTCGAAACTTCTCCGCAGTGAAAGATAAATGATC*TTGACTGATTTTTCCATGGA*GTTTTAGAGCTAGAAATAGCAAGTTAAAATAAG	pROS12 + ScURA3 gRNA
8553	TGCCCAGTATTCTTAACCCAACTGCACAGAACAAAAACCTGCAGGAAACGAAGATAAATCAAAACTGTATTATAAGTAAATGCATGTATACTAAACTCACAAATTAGAGCTTCAATTTAA	Repair DNA ScURA3
8554	TTAAATTGAAGCTCTAATTTGTGAGTTTAGTATACATGCATTTACTTATAATACAGTTTTGATTTATCTTCGTTTCCTGCAGGTTTTTGTTCTGTGCAGTTGGGTTAAGAATACTGGGCA	Repair DNA ScURA3
9310	TCGCCTGCAAATCGTCATCG	Diagnostic primer ILV6
9311	CCTTAGAAACATCCGAGCTCCTCCTGGGCCTCTATACATC	Repair fragment construction
9312	GATGTATAGAGGCCCAGGAGGAGCTCGGATGTTTCTAAGG	Repair fragment construction
9313	AGCTGGTCGCCAAGGACTAC	Diagnostic primer ILV6
9314	CTACTGCGCCAATTGATGAC	Diagnostic primer ScURA3
9317	GCCCTACACGTTCGCTATGC	Diagnostic primer ScURA3
9318	GTTGACACAGTCCGTGAAAC	Diagnostic primer SeURA3
9321	GGCGCATTGGAGTCAATGAG	Diagnostic primer SeURA3
9390	ATGGATTATAAAGATGACGATGACAAAC	Amplification cas9
9391	CCGCTCAGACCTTTCTCTTC	Amplification cas9
9392	TTTTGTATAACTAAATAATATTGGAAACTAAATACG	Amplification ScPHO5t
9393	**TGCCGAACTTTCCCTGTATGAAGCGATCTGACCAATCCTTTGCCGTAGTTTCAACGTATG**TTTTCATTTTTGCGATGCCAG	Amplification ScPHO5t + addition SHR F
9394	TGTTGATTATGTTTTTAAGAACTACTCAGAATG	Amplification AaTEF1p
9395	AGGCCAGGAACCGTAAAAAG	pUC19 backbone
9396	ATTTCATTCTGAGTAGTTCTTAAAAACATAATCAACAATGGATTATAAAGATGACGATGACAAACCTCCAAAAA	LCR bridging oligo
9397	TGACCCTCCAAAAAAGAAGAGAAAGGTCTGAGCGGTTTTGTATAACTAAATAATATTGGAAACTAAATAC	LCR bridging oligo
9398	TTCATACAGGGAAAGTTCGGCAGGTTTCTTAGACGTCAGGTGGC	LCR bridging oligo
9399	CCTTTTTACGGTTCCTGGCCTCACCTTTCGAGAGGACGATG	LCR bridging oligo
9663	**CATACGTTGAAACTACGGCAAAGGATTGGTCAGATCGCTTCATACAGGGAAAGTTCGGCA**TCAACATCTTTGGATAATATCAGAATGAG	Amplification panARSopt + addition of SHR F
10426	AATCTATAATCAGTCCATAGTCAACAAGAGCC	Amplification AaTEF1p
10427	TTTTCATTTTTGCGATGCCAGTTCTTTG	Amplification ScPHO5t
10432	AAAAACGCCAGCAACGCGGCCTTTTTACGGTTCCTGGCCTGCGGCCGCGCTTCCAGGTTTGGCACTGTC	40 bp to pUC19 + NotI restriction + Left Homology arm fw
10433	ACTTTGAGGGCTCTTGTTGACTATGGACTGATTATAGATTTACGAAGGCACTTTGCATGGG	Left Homology arm rv + 40 bp to AaTEF1p
10434	GACAACACCTGTTGTAATCGAGCTCTCGAGAACCCTTAATGCGCGACATCAAATACCTTTGTCC	40 bp to *natNT2* + right homology arm fw
10435	CACATTTCCCCGAAAAGTGCCACCTGACGTCTAAGAAACCGCGGCCGCACGACGCGGACGAAGAGAAAG	Right homology arm rv + NotI restriction + 40 bp to pUC19
10436	ATAAGGGAAACTCAAAGAACTGGCATCGCAAAAATGAAAATAGGTCTAGAGATCTGTTTAGC	40 bp to ScPHO5t + *natNT2* fw
10686	GAGTAAAGAAGCTCATCATTTATATAGATACGTTATGTAGATGTATAGAGGCCCAGGAGGGAGCTCGGATGTTTCTAAGGCTCTGTATGTACAAACTACGTATGTGACTTATACATTGCT	Repair DNA ILV6
10687	AGCAATGTATAAGTCACATACGTAGTTTGTACATACAGAGCCTTAGAAACATCCGAGCTCCCTCCTGGGCCTCTATACATCTACATAACGTATCTATATAAATGATGAGCTTCTTTACTC	Repair DNA ILV6
10992	GTTCAAGATGAATGTCTTGTCAAGATGATACAGAATGGGCATTCCCGGCGTATGGGATCTTCATGGCATCAAGCTTTTTCATTGGGTGTTTCTTCGACTAATGTGAAGGGAATGAACATT	Repair DNA SeATF1
10993	AATGTTCATTCCCTTCACATTAGTCGAAGAAACACCCAATGAAAAAGCTTGATGCCATGAAGATCCCATACGCCGGGAATGCCCATTCTGTATCATCTTGACAAGACATTCATCTTGAAC	Repair DNA SeATF1
10994	TTTGCTGTTTTGCGTAGGCAAAACATGTATTCGAATTTCGCTGTTTATGGGGAACTGAATAACGTTGGTGGTATGAACATGGACATGAGCGTAGTTCAGGGCACTCTACGGAATCGGGGC	Repair DNA SeATF2
10995	GCCCCGATTCCGTAGAGTGCCCTGAACTACGCTCATGTCCATGTTCATACCACCAACGTTATTCAGTTCCCCATAAACAGCGAAATTCGAATACATGTTTTGCCTACGCAAAACAGCAAA	Repair DNA SeATF2
10996	ATGAGAAAAATCAGGCCCCC	Diagnostic primer
10997	CTAAGGGCCTAAAAGGAGAGC	Diagnostic primer
10998	GAAGGATACGAACCACATATCACG	Diagnostic primer
10999	TAAAGCGACGCAAATTCGCC	Diagnostic primer
11000	CAGAAGAAAGCCAATTTAGCAG	Diagnostic primer
11001	TCAGGGATTTAAAAGCAGAGC	Diagnostic primer
11002	GGATAGTTTAGAGGAATACGAACCG	Diagnostic primer
11003	TATACGAGACCCGCGACG	Diagnostic primer

SHR sequences are shown in bold, gRNA sequences are shown in italics and digestion enzyme recognition sites are underlined

pUD423 was assembled from plasmids pCT, pUD528 and pUC19. The *Streptococcus pyogenes cas9* open reading frame (*cas9* ^D147Y P411T^ [36]) was amplified from the plasmid pCT (Addgene plasmid #60621) (https://www.addgene.org/) using the primers 9390 and 9391. The *AaTEF1* promoter flanked upstream by short homology flank (SHF) B was amplified from the plasmid pUD528 using the primers 3841 and 9394. The *ScPHO5* terminator fragment was amplified from pUD528 using the primers 9392 and 9393, resulting in the addition of SHR F downstream of the terminator. The three fragments together with the pUC19 backbone [37] amplified with the primers 7389 and 9395 were assembled in vitro using ligase chain reaction (LCR) with primers 9396–9399 as bridging oligonucleotides as described previously [38] and the resulting plasmid pUD423 was verified using digestion with *Nde*I.

The cassette for integration of *cas9* into the *SPR3* locus was assembled on pUD526. Flanks for homologous recombination of about 500 bp were amplified from genomic DNA of CBS1483 using primers sets 10432/10433 and 10434/10435 adding *Not*I restriction sites upstream of the left homology arm and downstream of the right homology arm and 40 bp homology flanks on both sides of the homology arms for “Gibson” assembly [39]. The *cas9* expression cassette was amplified from plasmid pUD423 using primers 10426 and 10427, the nourseothricin marker was amplified from pMEL15 [15] using primers 3597 and 10436 adding a 40 bp homology flank upstream of the *nat* gene, and the plasmid backbone was amplified from plasmid pUC19 using primers 7389 and 9395. Next, 0.2 pmol of each fragment were assembled into pUD526 using NEBuilder^®^ HiFi DNA Assembly Master Mix (New England BioLabs, Ipswich, MA), verified by digestion with BamHI and NotI. The integration cassette was obtained by digestion of the plasmid using NotI followed by gel purification.

**Fig. 2:**
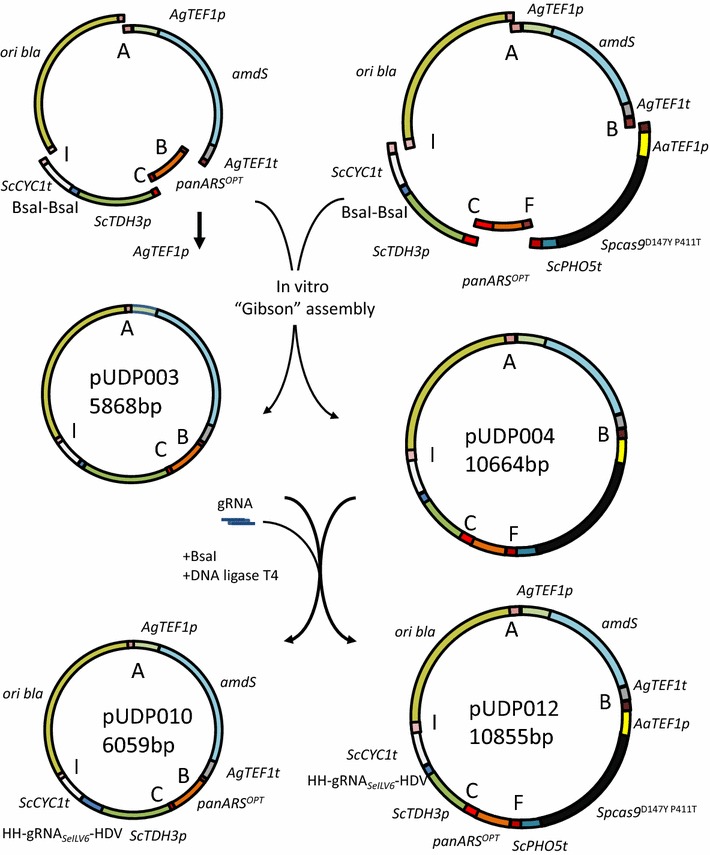
Construction of the gRNA expression plasmids pUDP003 and pUDP004. In vitro “Gibson” assembly [39] of functional parts containing an *amdS* selection marker cassette, a synthetic pangenomic yeast replication origin panARSopt, an *E. coli* replication origin from pBR322 and the *bla* gene conferring resistance to β-lactam antibiotics and a gRNA expression cassette using 60 bp synthetic homologous recombination sequences into pUDP003 and with the addition of a fragment carrying a *Spcas9* expression part into pUDP004. The ribozymes flanked gRNA is next directionally inserted into pUDP003 or pUDP004 using BsaI digestion and ligation yielding the gRNA expressing plasmids pUDP010 and pUDP012 respectively

pUDP003 was assembled from plasmids pUD527, pUD530, pUD531 and pUD532 (Fig. 2). The *amdS* selection cassette [34] was amplified from pUD527 using primers 3847 and 3276 containing SHF A and B flanks. The synthetic pangenomic yeast replication origin panARSopt [40] was amplified from pUD530 using primers 3841 and 3856 containing SHF B and C flanks. The gRNA introduction site was amplified from pUD531 using primer 3283 and 4068 containing SHF C and I flanks. The *E. coli* replication origin from pBR322 and the *bla* gene conferring resistance to β-lactam antibiotics were amplified from pUDP532 using primers 3274 and 3275 containing SHF I and A flanks. The amplified fragments were digested with DpnI, gel purified and quantified using a NanoDrop 2000 spectrophotometer (Thermo Fisher Scientific). 0.2 pmol of each fragment were assembled into pUDP003 using NEBuilder^®^ HiFi DNA Assembly Master Mix (New England BioLabs). The resulting plasmid pUDP003 was verified by restriction analysis using SspI.

pUDP004 was assembled from plasmids pUD423, pUD527, pUD530, pUD531 and pUD532 (Fig. 2). The *amdS* selection cassette [34] was amplified from pUD527 using primers 3847 and 3276 introducing SHF A and B flanks. The *cas9* expression cassette was amplified from pUD423 using primers 3841 and 9393 containing SHF B and F flanks. The synthetic pangenomic yeast replication origin panARSopt [40] was amplified from pUD530 using primers 9663 and 3856 containing the SHF C flank and introducing the SHF F flank, thereby replacing the SHF B flank. The gRNA introduction site was amplified from pUD531 using primers 3283 and 4068 containing SHF C and I flanks. The *E. coli* replication origin from pBR322 and the *bla* gene conferring resistance to β-lactam antibiotics were amplified from pUDP532 using primers 3274 and 3275 containing SHF I and A flanks. The amplified fragments were digested with DpnI, gel purified and quantified using a NanoDrop 2000 spectrophotometer (ThermoFischer Scientific). 0.2 pmol of each fragment were assembled into pUDP004 using NEBuilder^®^ HiFi DNA Assembly Master Mix (New England BioLabs). The assembled plasmid pUDP004 was verified by restriction analysis using PdmI.

The gRNA sequences for pUDP type plasmids were designed such that they could be synthesized and inserted into pUDP003 or pUDP004 by digestion with BsaI and ligation. From 5′ to 3′, the sequences were composed of a BsaI recognition site yielding correct sticky ends “GGTCTCGCAAA”, followed by the hammerhead ribozyme with the first six nucleotides being the reverse complement (^c^) of the first six nucleotides of the gRNA spacer “^c^N_6_^c^N_5_^c^N_4_^c^N_3_^c^N_2_^c^N_1_CUGAUGAGUCCGUGAGGACGAAACGAGUAAGC UCGUC”, followed by the 20 nucleotide gRNA spacer designed as previously [15], followed by the structural gRNA “GUUUUAGAGCUAGAAAUAGCAAGUUAAAAUAAGGCUAGUCCGUUAUCAACUUGAAAAAGUGGCACCGAGUCGGUGCUUUU”, followed by the Hepatitis Delta Virus ribozyme “GGCCGGCAUGGUCCCAGCCUCCUCGCUGGCGCCGGCUGGGCAACAUGCUUCGGCAUGGCGAAUGGGAC”, followed again by a BsaI recognition site yielding correct sticky ends “ACAGCGAGACC”. For multiplexing, linker “ACAGCGCAAA” was added between the HDV ribozyme of the first gRNA and the HH ribozyme of the second gRNA. Plasmids pUD536, containing the gRNA sequence targeting *SeILV6*, and pUD573, containing a polycistronic array with gRNAs targeting *SeATF1* and *SeATF2*, were de novo synthesised at GeneArt (Thermo Fisher Scientific). The plasmid pUDP010, expressing gRNA_*SeILV6*_, was constructed in a one-pot reaction by digesting pUDP003 and pUD536 using BsaI and ligating with T4 ligase. Similarly pUDP012, expressing gRNA_*SeILV6*_ and *Spcas9* ^D147Y P411T^, was assembled from pUDP004 and pUD536 and pUDP044, expressing gRNA_*SeATF1*_::gRNA_*SeATF2*_ and *Spcas9* ^D147Y P411T^ was assembled from pUDP004 and pUD573. Correct assembly of pUDP010 was verified by restriction analysis with SspI and correct assembly of pUDP012 and pUDP044 was verified by restriction analysis using PdmI. Plasmid pUDR107, expressing gRNA_*URA3*_, was constructed using NEBuilder^®^ HiFi DNA Assembly Master Mix by assembling the 2 μm fragment amplified from pROS12 with primer 8314 and the plasmid backbone amplified from pROS12 with primer 6005 as previously described in [15].

Plasmids pUDP003, pUDP004, pUDP010, pUDP012 and pUDP044 were deposited at addgene (http://www.addgene.org/) (Table 1).

#### Strain construction

The strain IMX1187 was constructed by transforming CBS1483 with 1 µg of the NotI-digested and gel purified integration cassette from pUD526 by electroporation and plated on YPD with nourseothricin (Fig. 1). After 5 days, 14 colonies had grown and integration of *cas9* was confirmed using primers 3750 and 9394. One of the colonies was stocked and sequenced.

IMX1205 (Fig. 1) was constructed by transforming IMX1187 by electroporation with 500 ng of pUDP010 and 1 µg of a 120 bp repair fragment obtained by mixing an equimolar amount of primers 10686 and 10687. Transformants were selected on SM-Ac plates. Transformants were confirmed using primers 9310 and 9313. Prior stocking the isolate was successively streaked out on SM-Ac, YPD and SM-FAc plates. Genotype was systematically verified after each plating round with primers 9310 and 9313. In the end, one of the colonies was stocked.

IMK771 (Fig. 1) was constructed by transforming CBS1483 by electroporation with 200 ng of pUDP012 and 1 µg of 120 bp repair product obtained by mixing an equimolar amount of primers 10686 and 10687. Transformants were selected on SM-Ac plates. Deletion of Se*ILV6* was confirmed using primers 9310 and 9313. Prior stocking the isolate was successively streaked out on SM-Ac, YPD and SM-FAc plates. Genotype was systematically verified after each plating round with primers 9310 and 9313. In the end, one of the colonies was stocked and sequenced.

IMK786 (Fig. 1) was constructed by transforming CBS1483 by electroporation with 200 ng of pUDP044 and 1 µg of 120 bp repair product obtained by mixing an equimolar quantity of primers 10992 and 10993 for *SeATF1* and 1 µg of 120 bp repair product obtained by mixing an equimolar quantity of primers 10994 and 10995 for *SeATF2*. Transformants were selected on SM-Ac plates, deletion of *SeATF1* and *SeATF2* was confirmed using primers 11000/11001 and primers 11002/11003, respectively. Prior stocking the isolate was successively streaked out on SM-Ac, YPD and SM-FAc plates. Genotype was systematically verified after each plating round with primers pairs 11000/11001 and 11002/11003 to confirm *SeATTF1* and *SeATF2* deletions. In the end, one of the colonies was stocked.

IMK813 (Fig. 1) was constructed by transforming Weihenstephan 34/70 by electroporation with 200 ng of pUDP044 and 1 µg of 120 bp repair product obtained by mixing an equimolar quantity of primers 10992 and 10993 for *SeATF1* and 1 µg of 120 bp repair product obtained by mixing an equimolar quantity of primers 10994 and 10995 for *SeATF2*. Transformants were selected on SM-Ac plates, deletion of *SeATF1* and *SeATF2* was confirmed using primers 11000/11001 and primers 11002/11003, respectively.

### Next generation sequencing

IMX1187 and IMK771 were incubated in 500-mL shake-flasks containing 100 mL liquid YPD medium at 20 °C on an orbital shaker set at 200 rpm until the strains reached stationary phase with an OD_660_ between 12 and 20. Genomic DNA for whole genome sequencing was isolated using the Qiagen 100/G kit (Qiagen, Hilden, Germany) according to the manufacturer’s instructions and quantified using a Qubit^®^ Fluorometer 2.0 (ThermoFisher Scientific). 51.57 µg of genomic DNA from IMX1187 and 14.20 µg from IMK771 was sequenced by Novogene Bioinformatics Technology Co., Ltd (Yuen Long, Hong Kong) on a HiSeq 2500 (Illumina, San Diego, CA) with 150 bp paired-end reads using True-seq PCR-free library preparation (Illumina). CRISPR-Cas9 assisted deletions were verified by mapping the sequencing reads onto the *S. pastorianus* CBS1483 genome [4] using the Burrows–Wheeler Alignment tool (BWA) and further processed using SAMtools [41, 42]. The deletions were confirmed by visualising the generated .bam files in the Integrative Genomics Viewer (IGV) software [43]. The sequencing data are available at NCBI (https://www.ncbi.nlm.nih.gov/) under the Bioproject PRJNA397648.

## Results

### Construction of a *S. pastorianus* strain expressing *cas9*

**Fig. 3:**
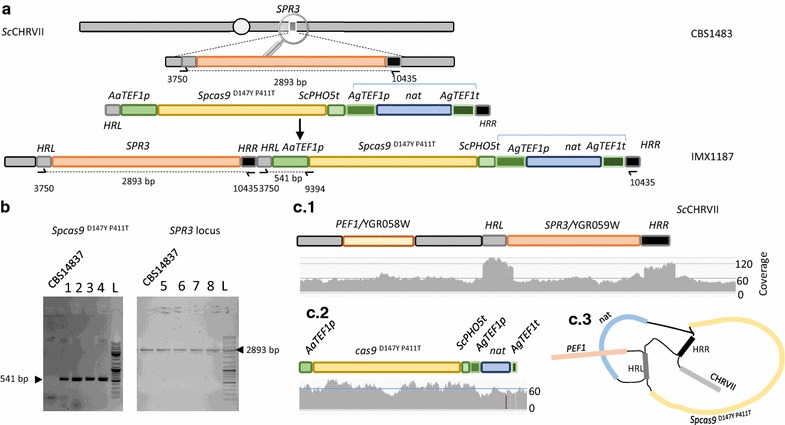
Integration of *Spcas9* ^D147Y P411T^ at the *SPR3* locus in *S. pastorianus* CBS1483. **a** Schematic representation of the integration of *Spcas9* ^D147Y, P411T^ [36] and the *nat* selection marker. The integration is directed by homology regions of 480-bp (HRL) and 506-bp (HRM) to complete the double cross over integration. **b** Verification of the construction of strain IMX1187. Presence of the integration fragment carrying *Spcas9* and *nat* genes and of the *SPR3* open reading frame and was checked in four transformants with primers 3750 and 9394 (lanes 1–4) and with primers 3750 and 10435 (lanes 5–8) respectively. The strain host strain CBS1483 was used as reference. The transformant in lanes 1 and 5 was renamed IMX1187. The lane labelled with L designated the position of the DNA ladder [Gene ruler DNA ladder Mix (ThermoFischer Scientific #SM0332)]. **c** 1−Mapping of the 150 bp Illumina sequencing reads of IMX1187 onto the reference genome of CBS1483 [4] at the *SPR3* locus reveals about 120-fold coverage of the homology regions HRL and HRM while the average coverage is about 60-fold. 2—Mapping of the IMX1187 150 bp Illumina sequencing reads onto the integrated fragment containing the *Spcas9* and *nat* genes reveals about 60-fold coverage of the casette. 3—Assembly graph of IMX1187 mapping on *SPR3*, *Spcas9* and their paired reads using SPAdes [46]

To limit construct instability and facilitate successive genome editing events, a copy of the *Streptococcus pyogenes cas9* variant, *cas9* ^D147Y P411T^ [36] was integrated in the genome of *S. pastorianus* CBS1483. The *S. cerevisiae SPR3*/YGR059W gene is involved in sporulation: a function impaired in *S. pastorianus;* therefore it was chosen as integration site as the impact on growth of deletion of *SPR3* should be negligible. Additionally, *SPR3* is located in the middle of the right arm of the *S. cerevisiae* CHRVII which counts only one copy in CBS1483, which should enable stable integration of a single *cas9* copy [4]. To prevent off-target integration driven by homology of the promoter and terminator, *cas9* was placed under the control of the *TEF1* promoter from *Arxula adeninivorans,* which had been shown to be functional in *Saccharomyces* yeast [44]. The nourseothricin acetyl transferase expression cassette natNT2 expressed from the *TEF1* promoter from the yeast *Ashbya gossypii* was used as a marker to select for integration [45] (Fig. 3). To guide the chromosomal integration of the endonuclease construct, the *cas9* containing fragment was flanked with an homology region of 480-bp targeting the *SPR3* promoter region (HRL, Fig. 3a) and a 506-bp targeting the *SPR3* terminator region (HRM) to complete the double cross over integration (Fig. 3a). These elements were assembled into a transformation cassette on pUD526 and the purified integration fragment was used to transform *S. pastorianus* CBS1483 yielding 14 transformants. In comparison, the same transformation in the laboratory *S. cerevisiae* CEN.PK113-7D yielded 476 transformants. Both transformations were performed simultaneously and under identical experimental conditions, therefore the difference in obtained transformants reflected the strong resilience of industrial *S. pastorianus* strains to transformation. The presence of the integrated construct was confirmed in all four tested colonies by PCR using specific primers (3750 and 9394) which amplify between the left homology arm for *SPR3* and the end of the *AaTEF1* promoter. Unexpectedly, a PCR targeting the *SPR3* open reading frame using primers 3750 and 10435 yielded a fragment size corresponding to the wild type. Concomitantly, PCRs targeting *cas9* confirmed the integration in CHRVII in all four tested transformants, suggesting that either *SPR3* might have been duplicated prior to replacement of one of the copies by *cas9*, or the cassette was not integrated as intended. To resolve the recombined *SPR3* locus map, one of the transformants, was renamed IMX1187 and resequenced using Illumina technology. Mapping of the IMX1187 Illumina pair reads (2× 150 bp) on the CBS1483 reference genome sequence confirmed the presence of the *S. cerevisiae SPR3* wild type locus, but it also revealed that the region used for the integration HRL and HRM, exhibited a sequence depth coverage twofold higher than the *SPR3* open reading frame and the surrounding chromosomal region (Fig. 3c). In the meantime, mapping of the IMX1187 reads on the sequence of the deletion cassette including the *cas9* and *nat* genes confirmed the single integration of the transformed fragment. Additionally, absence of reads mapping the β-lactamase gene *bla* present on pUD526 excluded the possibility that the plasmid got mistakenly integrated in the genome. To demonstrate anchoring of the cassette into CHRVII, the reads that mapped to the *SPR3* region and to the integration fragment containing *cas9* and *nat* (including corresponding paired reads) were extracted and assembled using SPAdes [46]. The assembly confirmed that the cassette was anchored in CHRVII and the obtained graph suggested that the *cas9/nat* cassette integrated by single crossover resulting in a duplication of the integration site HMR or HML and integration of the *cas9* cassette (Fig. 3). However, the integration cassette was fully integrated and should result in expression of Cas9.

**Fig. 4:**
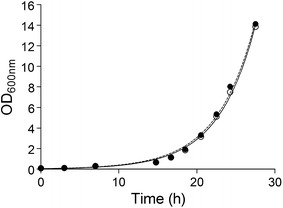
Growth curve of the *S. pastorianus* strains CBS1483 (filled circle) and IMX1187 (*Spcas9*) (open circle). The strains CBS1483 and IMX1187 were strains were grown in complex medium (YPD) at 20 °C. Growth was monitored based on optical density at 660 nm (OD_660_) measurements. The data plotted are average and standard deviation of three biological replicates

In literature, there are conflicting reports about the physiological consequences of Cas9 expression in *Saccharomyces cerevisiae,* depending on the mode and tuning of expression of the endonuclease gene [15, 19, 47]. Therefore, the growth rates of the *S. pastorianus* CBS1483 and IMX1187 (*AaTEF1*-*cas9*) were measured in YPD at 20 °C. The average maximum specific growth rate derived for biological triplicates for both strains did not deviate more than 2%. The strains CBS1483 and IMX1187 exhibited growth rate of 0.263 ± 0.002 and 0.258 ± 0.001 h^−1^ respectively (Fig. 4). This result confirmed that single integration of *cas9* in CBS1483 (IMX1187) did not significantly affect the maximum specific growth rate.

#### gRNA delivery systems for efficient editing in *S. pastorianus*

**Fig. 5:**
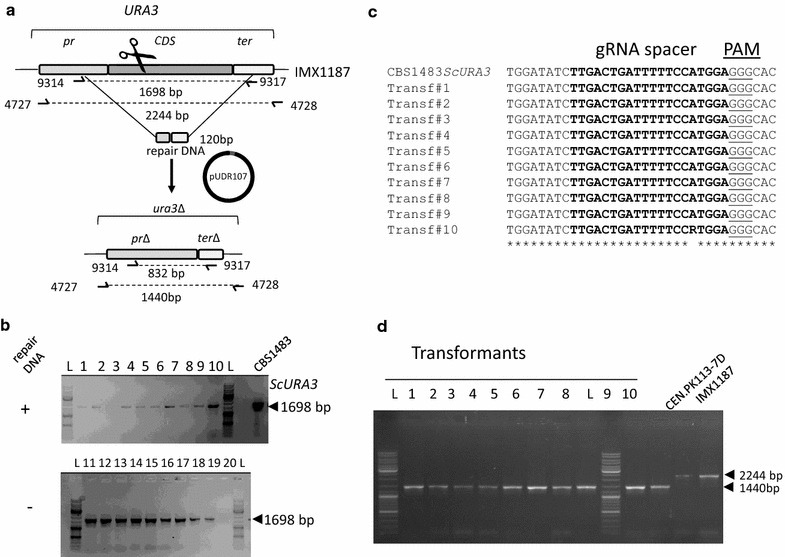
Deletion of *ScURA3* in IMX1187 and IMX585 using RNA III polymerase dependent (*SNR52p*) gRNA expression. **a** Representation of the native and deleted *ScURA3*. The plasmid pUDR107 carried a gRNA under the control of the *SNR52p*. Primers used for validation of the deletion are indicated. **b** Validation of transformants of the *S. pastorianus* IMX1187 strain with pUDR107 in presence or not of a 120 bp repair DNA. The PCR reactions were performed with the primers 9314 and 4728. All lanes (1–20) showed a PCR product of 1698 bp corresponding to the wildtype allele. The lane labelled with L designated the position of the DNA ladder [Gene ruler DNA ladder Mix (ThermoFischer Scientific #SM0332)]. **c** Sanger sequencing results of purified PCR fragments of ten transformants derived from the transformation of IMX1187 with pUD107 (gRNA_*URA3*_). The gRNA spacer used to direct Cas9 is indicated in bold and the PAM sequence is underlined. **d** Validation of transformants of the *S. cerevisiae* IMX585 strain with pUDR107 in presence or not of a 120 bp repair DNA. The PCR reactions were performed with the primers 4727 and 4728. The lanes (1–10) corresponding to transformants obtained with repair DNA showed a PCR product of 1440 bp corresponding to the deleted allele. The control lane labelled CEN.PK113-7D showed the wild type fragment at 2244 bp. The lane labelled with L designated the position of the DNA ladder

After establishing the chromosomal integration of *cas9* in the genome of CBS1483, the next step consisted in demonstrating the activity of the RNA-programmed endonuclease. To do so, two gRNA delivery systems were tested, one based on the existing RNA polymerase III dependent system developed for *S. cerevisiae* [15] and one expression system based of ribozyme flanked gRNA expressed from a RNA polymerase II promoter. Firstly, the deletion of *URA3* using the traditional RNA polymerase III system was tested in *S. pastorianus* strain IMX1187 (*AaTEF1p*-*cas9*). The selected 20-bp spacer to target *URA3* matched the *ScURA3* allele sequence perfectly (TTGACTGATTTTTCCATGGA), but carried one mismatch on the 12th position from its 3′end (TTGACTGACTTTTCCATGGA) compared to the *S. eubayanus* allele (*SeURA3*). Both alleles shared the same gRNA spacer adjacent motif (PAM) sequence (GGG) and CBS1483 harbored three *S. cerevisiae* and two *S. eubayanus* alleles. The gRNA_*URA3*_ was expressed by the RNA polymerase III dependent promoter *SNR52p* [16, 48] from the pROS12 plasmid, which carries a hygromycin resistance marker *hph* [15]. The resulting plasmid pUDR107 (gRNA_*URA3*_) was transformed in IMX1187 alone or together with two 120 bp double stranded repair DNA fragments for *ScURA3 and SeURA3*. In absence of repair DNA, the transformation of the *URA3* gRNA should in theory be lethal and yield few to no transformants, due to the inefficiency of non-homologous end joining (NHEJ) (Fig. 5a). However, the transformation of IMX1187 with pUDR107 alone returned several hundred of colonies, a number comparable to when the repair DNA was also provided. A set of ten clones from each transformation were picked and their genotype was diagnosed by specific PCR (9314 and 9317 for *ScURA3* and 9318 and 9321 for *SeURA3*). All transformants either derived from the transformation with or without supply of a repair DNA produced a band with a size compatible with the wild type allele (Fig. 5b). The Sanger sequencing results of the amplified fragments showed no indels at the site of the anticipated cut. With the exception of clone #10 that showed an unresolved purine (R), all *URA3* sequences were identical to that of the reference IMX1187, confirming the absence of editing (Fig. 5c). Therefore, to exclude defective expression of the gRNA, pUDR107 (gRNA_*URA3*_) was also transformed in *S. cerevisiae* IMX585 (*cas9*) [15] together with the *ScURA3* 120 bp repair DNA. Out of the couple of dozens transformants, ten were randomly picked and diagnosed with by PCR. All transformants exhibited a band at 1440 bp characteristic of the *URA3* deletion. The same PCR from the untransformed CEN.PK113-7D yielded a fragment of 2244 bp (Fig. 5d). This result established that pUDR107 enabled functional Cas9-mediated gene editing in *S. cerevisiae* IMX585, but not in *S. pastorianus* IMX1187.

**Fig. 6:**
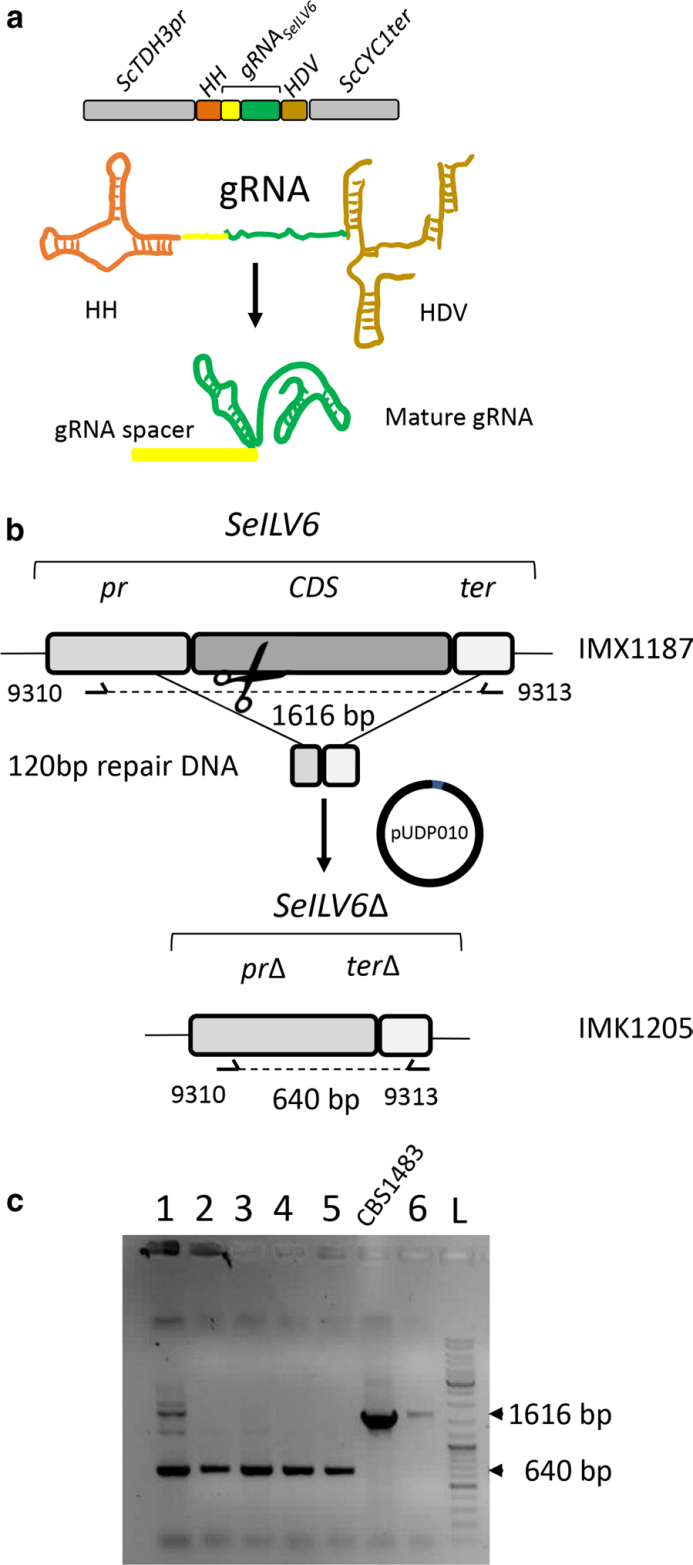
Ribozymes flanked gRNA driven deletion of *SeILV6* in *S. pastorianus* IMX1187. **a** Representation of the gRNA expression cassette in pUDP010. The *gRNA*_*SeILV6*_ was flanked on its 5′ by a hammerhead ribozyme (HH represented in orange) and on its 3′ by a hepatitis delta virus (HDV represented in bronze) ribozyme. This construct was under the control of the RNA polymerase II promoter *ScTDH3* and the *ScCYC1* terminator. Upon ribozyme self-cleavage, a mature gRNA comprising the *SeILV6* guiding spacer (in yellow) and the constant structural gRNA fragment (in green) is released. **b** Schematic representation of the *SeILV6* editing upon transformation of IMX1187 with pUDP010. The primers for the validation of transformants are indicated. **c** Validation of transformants of the *S. pastorianus* IMX1187 strain with pUDP010 in presence of a 120 bp repair DNA. The lanes (1–5) corresponding to the transformants obtained with repair DNA showed a PCR product of 640 bp corresponding to the deleted allele. One of the transformants exhibiting an *SeILV6* deletion was renamed IMK1205. The control lane labelled CBS1483 and lane 6 corresponding to one transformant obtained without repair DNA showed a PCR product corresponding to the wild type fragment at 1616 bp. The lane labelled with L designated the position of the DNA ladder [Gene ruler DNA ladder Mix (ThermoFischer Scientific #SM0332)]

While gRNA transcript level was not measured, RNA polymerase III expression is known to be low [49], a level which might be insufficient to enable efficient Cas9-mediated introduction of a DSB. To circumvent this and to ensure high expression of the Cas9 programming RNA. In this approach, the gRNA was placed behind the control of the constitutive *ScTDH3* promoter. To prevent modifications inherent to RNA polymerase II transcribed RNA, the gRNA was flanked by a Hammerhead ribozyme (HH) and a Hepatitis Delta Virus ribozyme (HDV) on its 5′ and 3′ end respectively [21] (Fig. 6a). After transcription and self-cleavage of both ribozymes, high transcript levels of mature gRNA should be possible. Such an expression system was constructed, resulting in plasmid pUDP003, which harbored the *S. cerevisiae* codon optimized *Aspergillus nidulans* acetamidase gene (*amdS*) [34] and enabled insertion of a specific gRNA. This strategy was tested by attempting deletion of the *SeILV6* gene in IMX1187 (*AaTEF1p*-*cas9*). The *S. pastorianus* strain CBS1483 and IMX1187 harbored only one *ILV6* gene that originates from the *S. eubayanus* sub-genome [4]. The *SeILV6* gene is located on the SeCHRIII, a chromosome present in four copies [4]. The gRNA_*SeILV6*_ was inserted in plasmid pUDP003 (Fig. 2), resulting in plasmid pUDP010 (HH-gRNA_*SeILV6*_-HDV *amdS*). Despite the absence of a *S. cerevisiae ILV6* allele in IMX1187, the gRNA_*SeILV6*_ was designed to target *ILV6* in *S. cerevisiae* as well. Thus, prior testing pUDP010 in *S. pastorianus*, the plasmid was transformed in *S. cerevisiae* IMX585. In the absence of a repair fragment, only 10 transformants were obtained while more than 500 were obtained when the repair fragment was included. Eventually a diagnostic PCR using specific primers confirmed successful deletion of *ILV6* in IMX585 for all tested colonies. Similarly, transformation of pUDP010 (HH-gRNA_*SeILV6*_-HDV *amdS*) in *S. pastorianus* IMX1187 (*AaTEF1p*-*cas9*) yielded 18 transformants when a 120 bp repair fragment was co-transformed against just one when the repair fragment was omitted. Diagnostic PCR using primers 9310 and 9313 confirmed successful deletion of Se*ILV6* in IMX1187 for all tested colonies (Fig. 6c). It should be noted that the absence of bands of original size confirmed that all four copies of *SeILV6* were deleted. The PCR characterization of the unique transformant obtained in absence of repair DNA indicated that the *ILV6* locus was not deleted, since a band with a size compatible with the reference length was amplified, suggesting that the CRISPR-Cas9 induced DSB was repaired by NHEJ (Fig. 6c).

The ability to obtain successful deletion of *ILV6* using the pUDP expression system indicated effective expression of the integrated *cas9* in *S. pastorianus* IMX1187, despite its imperfect integration in the *SPR3* locus. The failure to obtain deletion of *URA3* using the RNA polymerase III dependent gRNA expression system in *S. pastorianus* IMX1187 while deletion was possible in *S. cerevisiae* IMX585 indicated that this gRNA expression system was not effective in *S. pastorianus*. Based on literature, this ineffectiveness may be caused by low gRNA transcripts levels. Regardless, the new pUDP expression system was functional in *S. pastorianus* and the deletion of *ILV6* constituted the first reported successful use of Cas9 engineering in *S. pastorianus*.

#### Plasmid-based co-expression of Cas9 and gRNA in *S. pastorianus*

Given the notoriously low efficiency of gene insertion by homologous recombination in the genome of *S. pastorianus*, a plasmid was designed for co-expression of *cas9* together with the gRNA, which would render *cas9* expression more reproducible and facilitate genome editing in different *S. pastorianus* strains. The plasmid pUDP004 combined the *cas9* expression cassette previously integrated in IMX1187 and the different elements of pUDP003 including the RNA polymerase II dependent gRNA expression cassette (Fig. 2). To assess the efficacy of the pUDP004 system relative to the chromosome borne *cas9* together with the pUDP003 system, gRNA_*SeILV6*_ was inserted in pUDP004 and the resulting plasmid pUDP012 was used to transform CBS1483. In absence of a 120-bp repair DNA, a total of 14 transformants were obtained, while the number of transformants increased by 63-fold reaching a total of 884 transformants when the repair fragment was co-transformed. Diagnostic PCR using primers 9310 and 9313 confirmed successful deletion of Se*ILV6* in for all tested colonies and one colony producing a fragment corresponding to effective deletion of *SeILV6* was stocked as IMK771. To eliminate any doubt, the IMK771 genome was resequenced using Illumina sequencing technology. The 150-bp pair-end reads were mapped on the CBS1483 reference genome sequence [4] and as expected no reads mapped to the region targeted for deletion, indicating complete deletion of all four alleles of *SeILV6*. These results demonstrated that the plasmid-based co-expression of *cas9* and a gRNA was functional and could be used for effective genome editing in *S. pastorianus*.

#### Multiplexing gene targeting by expression of double ribozyme flanked gRNAs array

Despite the preexisting good genetic accessibility of *S. cerevisiae* strains, CRISPR-Cas9 mediated editing greatly simplified genome engineering approaches. In particular, the ability to multiplex editing events [15, 18, 50]. Therefore, the possibility of multiplexed gRNA expression was investigated in the pUDP expression system. Conveniently, the self-cleaving properties of the ribozymes might be compatible with the construction of adjacent HH-gRNA-HDV linked in a polycistronic array.

**Fig. 7:**
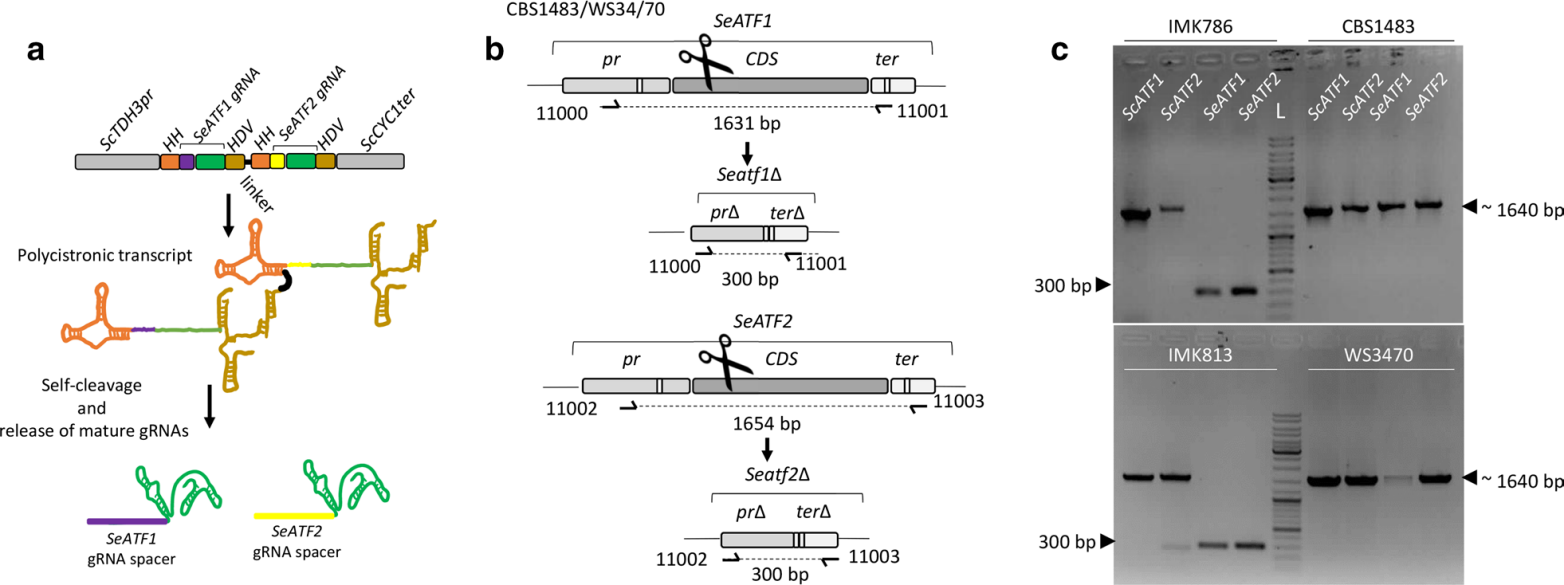
Simultaneous deletion of all *SeATF1* and *SeATF2* alleles using a single ribozymes flanked gRNA array in *S. pastorianus* CBS1483 and Weihenstephan 3470 (WS3470). **a** Representation of the gRNA array expression cassette in pUDP044. The dual gRNA array was under the control of the RNA polymerase II promoter *ScTDH3* and *ScCYC1* terminator. Each gRNA was flanked on its 5′ by a hammerhead ribozyme (HH represented in orange) and on its 3′ by a hepatitis delta virus (HDV represented in bronze) ribozyme and they were separated by a 10 bp linker. Upon ribozyme self-cleavage, the mature gRNAs are released. The *SeATF1* guiding spacer (in purple), the *SeATF2* guiding spacer (in yellow) and the constant structural gRNA fragment (in green) are indicated. **b** Schematic representation of the *SeATF1* and *SeATF2* editing upon transformation of CBS1483 with pUDP044. The primers for the validation of transformants are indicated. **c** Validation of transformants of the *S. pastorianus* CBS1483 strain with pUDP044 in presence of a 120 bp repair DNA. The PCR reactions were performed with the primers pairs 11000/11001 for *SeATF1* and 11002/11003 for *SeATF2*. The isolate renamed IMK786 exhibited bands at 300 bp corresponding to the deletions of *SeATF1* and *SeATF2*. *ScATF1* and *ScATF2* were amplified using the primer pairs 10996/10997 and 10998/10999 respectively and exhibited wild type length. Similarly, transformants resulting from the transformation of pUDP044 in presence of a 120 bp repair DNA were checked with the primers pairs 11000/11001 for *SeATF1* and 11002/11003 for *SeATF2.* The isolate renamed IMK813 exhibited bands at 300 bp corresponding to the deletions of *SeATF1* and *SeATF2*

Encouraged by the successful *SeILV6* deletion using pUDP004 based gRNA expression, a tandem array of [HH-gRNA-HDV] targeting *SeATF1* and *SeATF2* in *S. pastorianus* was designed. The two HH-gRNA-HDV were spaced with a 10-bp linker. The synthesized array was placed under the control of the *ScTDH3* promoter in pUDP004 as described earlier for the *SeILV6* gene. The recombinant plasmid pUDP044 (*amdS cas9 TDH3*p-HH-gRNA_*SeATF1*_-HDV-HH-gRNA_*SeATF2*_-HDV-*CYC1*t) was then used to transform two *S. pastorianus* strains: CBS1483 and Weihenstephan 34/70 (Fig. 7a). CBS1483 harboured one and three copies of *SeATF1* and *SeATF2* respectively, while Weihenstephan 34/70 missed one *SeATF2* allele relative to CBS1483. Co-transformation of CBS1483 and Weihenstephan 34/70 with pUPD044 and the corresponding repair fragments yielded 43 and 189 transformants per plate respectively. In the absence of repair fragments, 15 and 44 colonies were obtained in CBS1483 and Weihenstephan 34/70, respectively. A randomly picked set of seven colonies transformed with repair fragment were verified by PCR, which confirmed that all copies of *SeATF1* and *SeATF2* were deleted. One of the CBS1483 transformants exhibiting the correct double *SeATF1/SeAFT2* deletion was named IMK786 and similarly a Weihenstephan transformant was named IMK813 (Fig. 7). The designed gRNAs were also confirmed to be specific to the *S. eubayanus* genes as the *ScATF1* and *ScATF2* genes were not affected (Fig. 7c). To the best of our knowledge, this represents the first application of polycistronic ribozyme flanked gRNA, as well as the first demonstration of a successful double deletion in *S. pastorianus*.

## Discussion

### *Saccharomyces pastorianus* is not genetically amendable

The results reported in this study firmly established that CRISPR-Cas9 improves the performance of homology-directed recombination in *S. pastorianus*. In contrast to *S. cerevisiae,* a species amenable to genetic modification, the interspecific hybrid *S. pastorianus* has shown higher resilience to targeted genetic alterations. This was exemplified by the attempt to integrate the *cas9* gene at a specific chromosomal site using traditional double cross over. The size of the cassette complicated the genotype characterisation, but the presence of the endonuclease gene was confirmed and although whole genome resequencing of the strain IMX1187 did not completely resolve the structure of the recombined locus, it strongly suggested that a single crossover integration event occurred, resulting in integration of *cas9* next to *SPR3* instead of replacing *SPR3* as intended. Several literature reports corroborated our unfortunate experience [7, 8]. In different microbial systems, the efficiency of integration by homologous recombination was improved by impairing the non-homologous end-joining (NHEJ) function [51–53]. This approach, though successful, was often accompanied by side effects such as an exacerbated sensitivity to environmental stresses. In *S. cerevisiae*, inactivation of Yku70 and Yku80, two proteins involved in NHEJ, resulted in severe alterations of telomere maintenance and function as well as in deregulation of the cell cycle [54–58], which might explain why this strategy has never been attempted in *S. pastorianus*. Furthermore, the absence of improvement of the *S. pastorianus* genetic accessibility is not so surprising after all, since the brewing industry as most industries involved in fermentation of products intended for human consumption, has been reluctant to apply genetically modified organisms by fear of consumers group opinion [59], and has privileged classical strain improvement programmes.

Eventually, the results reported in this study demonstrated that the introduction of a DSB, which stimulates occurrence of homologous recombination, would represent an efficient solution to circumvent the natural resilience to targeted genetic modification in *S. pastorianus*.

### gRNA expression in *S. pastorianus*

**Fig. 8:**
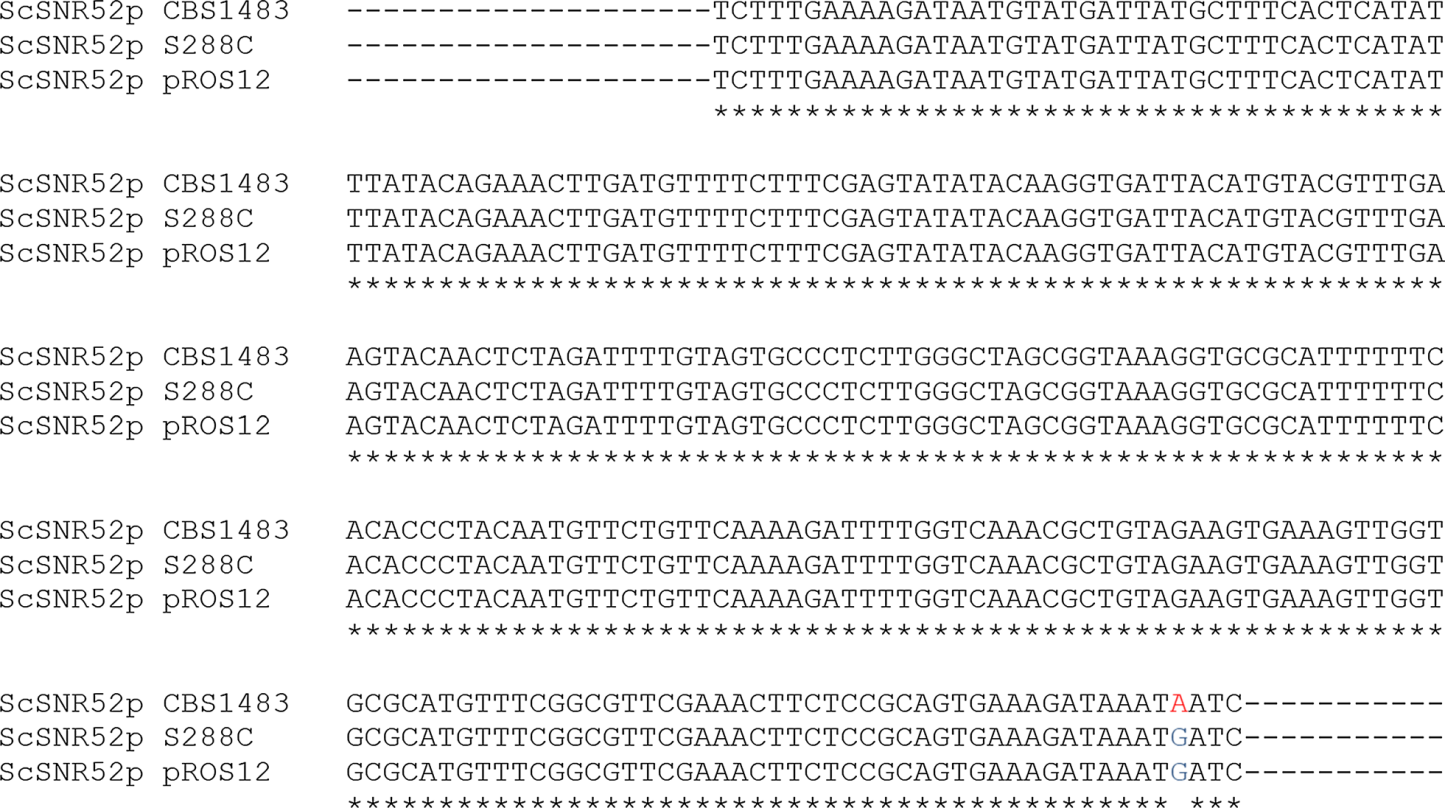
Sequence alignment of *ScSNR52* promoters derived from *S. cerevisiae* S288C [GenBank (http://www.ncbi.nlm.nih.gov/genbank/) Accession: NC_001137], pROS12 [Euroscarf (http://www.euroscarf.de/) Accession: P30789] [15] and *S. pastorianus* CBS1483 [Bioproject (http://www.ncbi.nlm.nih.gov/bioproject/) Accession: PRJNA266750] [4]. The sequences were obtained from XXX and aligned using Clustal W [69] with gap open penalty and gap extension penalty parameters set to 10 and 0.05 in the multiple alignment mode

Editing systems developed for *S. cerevisiae* could not be directly transfered to *S. pastorianus*. Although convoluted, the functionality of Cas9 in *S. pastorianus* was eventually demonstrated. In contrast to the situation in *S. cerevisiae*, the expression of the gRNA from the *SNR52* promoter was unsuccessful. While the objectives of the study were not to fully understand the origin of the lack of functionality of the *SNR52* driven gRNA expression, we could hypothesize that this problem might arise from the hybrid genome composition of *S. pastorianus*. Their alloaneuploid genome is a source of genetic innovations, e.g. increased chromosome copy number has facilitated introduction of allelic variations and cohabitation of the two parental genomes might have stimulated the adjustment of transcription circuits which together have contributed to adaptation of lager yeast to the intensified brewing environment [4, 60, 61]. Furthermore, many cellular functions are controlled by protein complexes which in hybrid strains may be formed by assemblies of subunits originating from both parental sub-genomes, thereby creating another source of variation [62]. The RNA polymerase III is a complex formed of six different subunits (*TFC1, 3, 4, 6, 7* and *8*) and the strain CBS1483 retained both parental gene sets [4]. Thus, the absence of editing might reflect a modification of the RNA polymerase III transcriptional control in *S. pastorianus* relative to *S. cerevisiae*. This could also be associated with promoter sequence variations between the parents and the hybrid. The inspection of the *SNR52* promoter sequences of the *S. cerevisiae* and *S. eubayanus* parents revealed nucleotide variations with S. *pastorianus* promoters (Fig. 8). The *ScSNR52* promoter from CBS1483 carried one mutation in position − 4 (G to A), while the CBS1483 *SeSNR52* promoter exhibited four single nucleotide variations with two located between the positions − 1 and − 100. In all configurations, the absence of editing points towards too low gRNA expression.

Fortunately, the proposed alternative involving expression of a ribozyme protected gRNA system turned out to be successful. In this method already used in human cells [63], plants [21, 32] and fungi [64, 65] the gRNA construct is expressed from a RNA polymerase II promoter. All CRISPR-Cas9 assisted deletions attempted (*SeILV6, SeATF1* and *SeATF2*) were introduced with high fidelity. The challenging nature of genetic modification in *S. pastorianus* does not come only from the low efficiency of homologous recombination, but also from the requirement to delete multiple alleles simultaneously due to its extensive aneuploidy [61]. In the case of *SeILV6*, four alleles were simultaneously deleted without introduction of any markers at the loci. The fact that all alleles were deleted at once as intended demonstrates the potency of a CRISPR-Cas9 induced DSB to stimulate targeted homology-mediated integration and circumvent unreliability of recombination in *S. pastorianus*. Remarkably, this could be achieved simultaneously at two different chromosomal loci (*SeATF1* on CHR SeVIII-SeXV and *SeATF2* CHR SeVII-ScVII) as well [4]. In total, this resulted in the deletion of five different alleles, one short to the highest number of simultaneously completed deletions in *S. cerevisiae* [15]. While previously suggested [21, 63], polycistronic ribozymes flanked gRNA expressed from a RNA pol II promoter had never been assayed before. Our results experimentally confirmed that 5′ and 3′ extension as designed at the junction of the two gRNA cassettes did not hinder self-cleavage of HH and HDV ribozymes and allow release of functional mature gRNAs. This result provided a glimpse of the potential of this mode of expression. It would suggest that construction of polycistronic array including more than two gRNA could be contemplated.

### Expanding the *S. pastorianus* genetic tool box

The present study delivered the first really efficient technical solution readily useable to perform targeted genetic modifications in *S. pastorianus*. The functionality of two modes of Cas9 expression was shown. Chromosomal integration of *cas9* (IMX1187) coupled with plasmid-based gRNA expression might be privileged when successive transformations are foreseen [7]. However, plasmid-based *cas9* and gRNA co-expression proved to be as effective and presents the advantage to be easily transferable in multiple strain backgrounds. For efficient use of the provided repair fragment to recombine at the locus of the Cas9-induced DSB, Cas9 activity and presence of the repair DNA have to be synchronous. The correct integration of the repair fragment during single and double gene editing showed that the endonuclease was transcribed and translated fast enough for free linear DNA to still be available for repair of the induced DSB. These outcomes were in line with similar approaches attempted in *S. cerevisiae* or in *Aspergillus niger* [36, 47, 64]. The presence of the gRNA is not constantly needed, as soon as the chromosomal double cut is inserted and preferably repaired, the plasmid has to be lost to recover a plasmid-free modified strain to either test the strain physiology or to prepare the constructed strain for a next editing round. The selection marker and replication origin used in the pUDP expression system tested in this work were designed to be broadly applicable and to facilitate rapid plasmid recycling. The dominant acetamidase marker confers the ability to use acetamide as sole nitrogen source and can be used in prototrophic strains such as lager yeasts or more generally industrial *Saccharomyces* strains. Plasmids carrying the *amdS* marker can be counter selected by growth in presence of fluoro-acetamide [34]. Additionally the panARSopt replication origin [40]) derived from *K. lactis* used in the pUDP expression system was shown to be functional in a wide range of yeast species including *S. cerevisiae*. Contrarily to most replication origins such as the 2 μm replication origin, which necessitates the presence of a wild type native 2 μm plasmid to provide the enzymatic replicative machinery, panARSopt does not require any other genetic element. Furthermore, like *ARS*-*CEN*-based plasmids, panARS-based plasmids showed loss frequencies ranging between 5 and 10% per generation when grown in non-selective conditions [40]. These properties should permit efficient use of the pUDP expression system in various strain backgrounds, which might help to standardize a genome editing protocol starting from the design and cloning of the gRNA to the selection of correctly edited strains which have lost the pUDP plasmid.

Finally, while the scope of this work limited the tools application to single and double gene deletions, the availability of CRISPR-Cas9 editing tool makes a broad range of genetic modifications possible. Analogously to modification techniques applied in *S. cerevisiae,* the pUDP expression system might be applied for in vivo site directed mutagenesis and targeted introduction of multiple genes or entirely new pathways. In *S. pastorianus*, such modifications would finally allow to systematically investigate the contribution of genes involved in brewing-relevant phenotypes of *S. pastorianus*. In particular, the use of subgenome specific gRNA targets could enable targeted modification of genes from the *S. cerevisiae* and *S. eubayanus* subgenomes and thereby enable research on their interaction. For example, elucidation of the role of individual flocculation genes or implication of individual maltose and maltotriose transporter in *S. pastorianus* could now be envisaged.

## Conclusions

The gRNA and Cas9 expression system developed in this study enabled CRISPR-Cas9 engineering in *S. pastorianus*. The system was applied successfully for the deletion of all alleles of *SeILV6* and could be multiplexed successfully to obtain the simultaneous deletion of all alleles of *SeATF1* and *SeATF2*. While the system was only tested for gene deletion in this study, functional CRISPR-Cas9 engineering in *S. pastorianus* should also facilitate approaches such as gene insertions and directed mutagenesis. As *S. pastorianus* is notoriously resilient to genetic modification, these developments significantly improve its genetic accessibility and facilitate future research into the complex allo-aneuploid genome of *S. pastorianus*.
